# Supplementary material for: Inter-Rater Reliability of Ergonomic Work Demands for Childcare Workers Using the Observation Instrument TRACK
Source: Int J Environ Res Public Health. 2020 Mar 2;17(5):1607. doi: 10.3390/ijerph17051607 (PMC7084378; doi:10.3390/ijerph17051607)
Supplement: Supplementary file 1 [file ijerph-17-01607-s001.pdf]

Supplementary File 1: Calculation of Gwet's AC_1

Gwet's AC_1 [1, 2] was calculated for each item as follows:

$$AC_1 = \frac{p\text{-}e(y)}{1\text{-}e(y)} \quad (1)$$

The range of the AC_1 is from -1 to 1, for complete disagreement and perfect agreement, respectively. The variable P is the ratio of agreement, as $N[A_{no},B_{no}]$ is 0 the form can be reduced to the following:

$$p = \frac{N[A_{yes}, B_{yes}] + N[A_{no}, B_{no}]}{N[Total]} = \frac{N[A_{yes}, B_{yes}]}{N[Total]} \quad (2)$$

The variable e(y) represents the chance agreement:

$$e(y) = 2q(1 - q)$$

The variable q is determined by the combined total of 'yes' registrations divided by the total number of registrations:

$$q = \frac{N[A_{yes}] + N[B_{yes}]}{N[Total]} = \frac{2 * N[A_{yes}, B_{yes}] + N[A_{yes}, B_{no}] + N[A_{no}, B_{yes}]}{N[Total]} \quad (3)$$

References

1. Gwet, K.L. Computing inter-rater reliability and its variance in the presence of high agreement. *Br. J. Math. Stat. Psychol.* **2008**, *61*, 29–48.
2. Gwet, K.L. *Handbook of inter-rater reliability: The definitive guide to measuring the extent of agreement among raters*, 4th ed; Advanced Analytics: Gaithersburg, MD, USA, 2014.
